# Retraction Note: Critical role of CCDC6 in the neoplastic growth of testicular germ cell tumors

**DOI:** 10.1186/s12885-021-07810-y

**Published:** 2021-01-27

**Authors:** Stefania Staibano, Gennaro Ilardi, Vincenza Leone, Chiara Luise, Francesco Merolla, Francesco Esposito, Francesco Morra, Maria Siano, Renato Franco, Alfredo Fusco, Paolo Chieffi, Angela Celetti

**Affiliations:** 1 Istituto di Endocrinologia ed Oncologia Sperimentale, CNRz, via S Pansini, 5, 80131 Naples, Italy; 2 Dipartimento di Medicina Molecolare e Biotecnologie Mediche, Università Federico II, Naples, Italy; 3 Dipartimento di Scienze Biomediche Avanzate, Università Federico II, Naples, Italy; 4 ISNT G. Pascale, Naples, Italy; 5 Dipartimento di Psicologia, Seconda Università di Napoli, 81100 Caserta, Italy

**Retraction Note: BMC Cancer (2013) 13:433**

**https://doi.org/10.1186/1471-2407-13-433**

The Editor has retracted this article [1] because a number of the figures exhibit irregularities. Specifically:

- In Figure 1B the blots in the ERK1/2 lanes for Testis and Interstitium appear to be the same
- In Figure 2E the blots in the CCDC6 lanes for shctrl-/+ and empty vector appear to be the same
- Figure 3 shows images labelled Seminoma and Embrionary carcinoma; however, these appear to represent the same tumour type

As these irregularities undermine the conclusions presented the Editor no longer has confidence in the article.

Angela Celetti, Stefania Staibano, Gennaro Ilardi, Francesco Merolla, Francesco Esposito, Francesco Morra, Renato Franco and Paolo Chieffi disagree with this retraction. Alfredo Fusco did not respond to correspondence about this retraction. The editor was not able to obtain a current email address for Vincenza Leone, Chiara Luise or Maria Siano.
